# Management of Iatrogenic External Iliac Artery Perforation With a Self-Made Covered Stent

**DOI:** 10.7759/cureus.28460

**Published:** 2022-08-27

**Authors:** Varun Marimuthu, Subramani Khamitkar Shankar Rao, Santosh Jadhav, Mohan H Nayak, Nagamani Alur

**Affiliations:** 1 Cardiology, Sri Jayadeva Institute of Cardiovascular Sciences and Research, Bangalore, IND

**Keywords:** covered stent, iliac artery, complications, haemorrhage, retroperitoneal space

## Abstract

Retroperitoneal hemorrhage (RPH) following transfemoral arterial access is a dreaded complication needing immediate management. A 70-year-old female with inferior wall myocardial infarction developed hemodynamic instability following transfemoral percutaneous coronary intervention. The evaluation revealed an RPH due to an iatrogenic guidewire-related perforation of the external iliac artery. This was successfully managed with the deployment of a custom, a self-made covered stent. In this report, we describe our method of creating and deploying this self-made stent and discuss potential issues compared to commercially available covered stent systems.

## Introduction

Vascular complications after trans-femoral arterial access (TFA) can range from local or expanding groin hematoma, arterio-venous fistula, and pseudoaneurysm to retroperitoneal hemorrhage (RPH) [1]. RPH is a rare, catastrophic complication following TFA. RPH causes severe lower abdominal and groin pain, hemodynamic collapse and death if not quickly detected and treated [2]. As most coronary interventions are now performed from trans-radial access (TRA), these vascular complications are encountered less. However, with the increasing use of left ventricular assist devices to support complex and high-risk interventions (CHIP) and transfemoral transcatheter aortic valve implantation (TAVI), TFA is as relevant as ever, and operators need to be cognizant of these complications, and their management.

In this report, we present a case of iatrogenic perforation of the right external iliac artery (EIA) during coronary intervention via TFA leading to life-threatening RPH. This was successfully managed with the implantation of a custom, self-made covered stent using readily available materials in the cardiac catheterization lab.

## Case presentation

A 70-year-old female with a past medical history of well-controlled diabetes mellitus, hypertension and the remote ischemic cerebrovascular accident was admitted. The patient had initially presented to an outside facility with inferior ST-elevation myocardial infarction (STEMI) and was transferred to our center after fibrinolytic therapy. At admission, her electrocardiogram (ECG) was repeated, and it showed good resolution of ST-T changes (>50% resolution). She was started on dual antiplatelets (DAPTs with aspirin and clopidogrel), statins and other supportive mediations. Anticoagulation with low-molecular-weight heparin (LMWH) was instituted. Transthoracic echocardiography (TTE) showed moderate left ventricular (LV) dysfunction (ejection fraction [EF] - 45%) with regional wall motion abnormalities (RWMA) in the right coronary artery territory with preserved wall thickness. Her lab parameters were all within normal limits.

**Figure 1:**
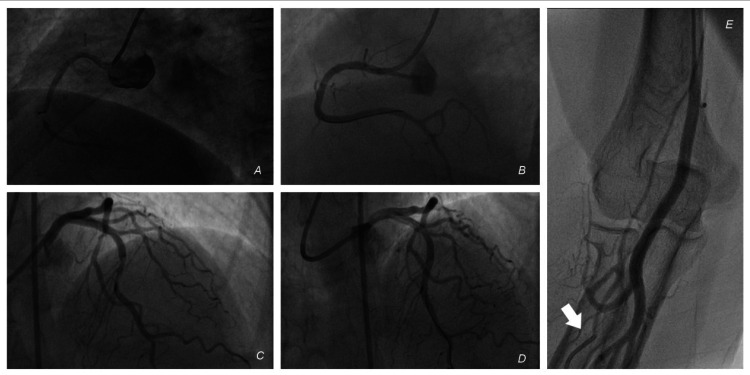
Trans-femoral percutaneous coronary intervention (A) RCA injection showing a mid-segment 90% lesion. (B) Post PCI. (C) LCA injection showing a mid-segment 80% lesion in the LAD. (D) Post PCI. (E) Radial artery sheath injection showing entry of the sheath into a small branch, precluding completion of the radial intervention. White arrow – a tip of the radial sheath. RCA – Right coronary artery, PCI - Percutaneous coronary intervention, LCA – Left coronary artery, LAD – Left anterior descending artery

The next day, she was taken up for a coronary angiogram (CAG). The right radial artery was punctured, but there was difficulty in advancing any guidewire (0.035” Teflon-coated wire and 0.021” glidewire (Terumo, Japan) beyond the mid-forearm. An angiogram done through the radial artery sheath revealed that the puncture was into a smaller branch (Figures 1A-1E, Supplementary Video 2). So, we decided to switch access to the femoral artery (FA). The right FA was punctured under fluoroscopic guidance in the first attempt. Diagnostic catheters were advanced from the femoral artery to the aorta through the right common iliac artery (CIA) without difficulty. CAG revealed the two-vessel disease. The left anterior descending artery (LAD) had a mid-segment 80% lesion, and the right coronary artery (RCA) had a mid-segment 80% lesion. The RCA was revascularized with a drug-eluting stent (DES) in the proximal vessel, with a good result. The Judkins right (JR) guide catheter was exchanged for an extra backup (EBU) catheter. A DES was deployed in the LAD with a good result. There was no use of GP2b3a inhibitors over the course of the percutaneous coronary intervention She was shifted to the coronary care unit (CCU) in a stable condition. An hour later, the patient complained of diffuse lower abdominal pain and was in visible distress. She had tachycardia and a dramatic drop in blood pressure to 80/50 mmHg. The puncture site at the right groin had no visible hematoma. With a strong suspicion of retroperitoneal bleeding, she was shifted back to the Cathlab. Bedside TTE did not reveal any mechanical complications like pericardial effusion and showed no new worsening of LV function.

**Figure 2:**
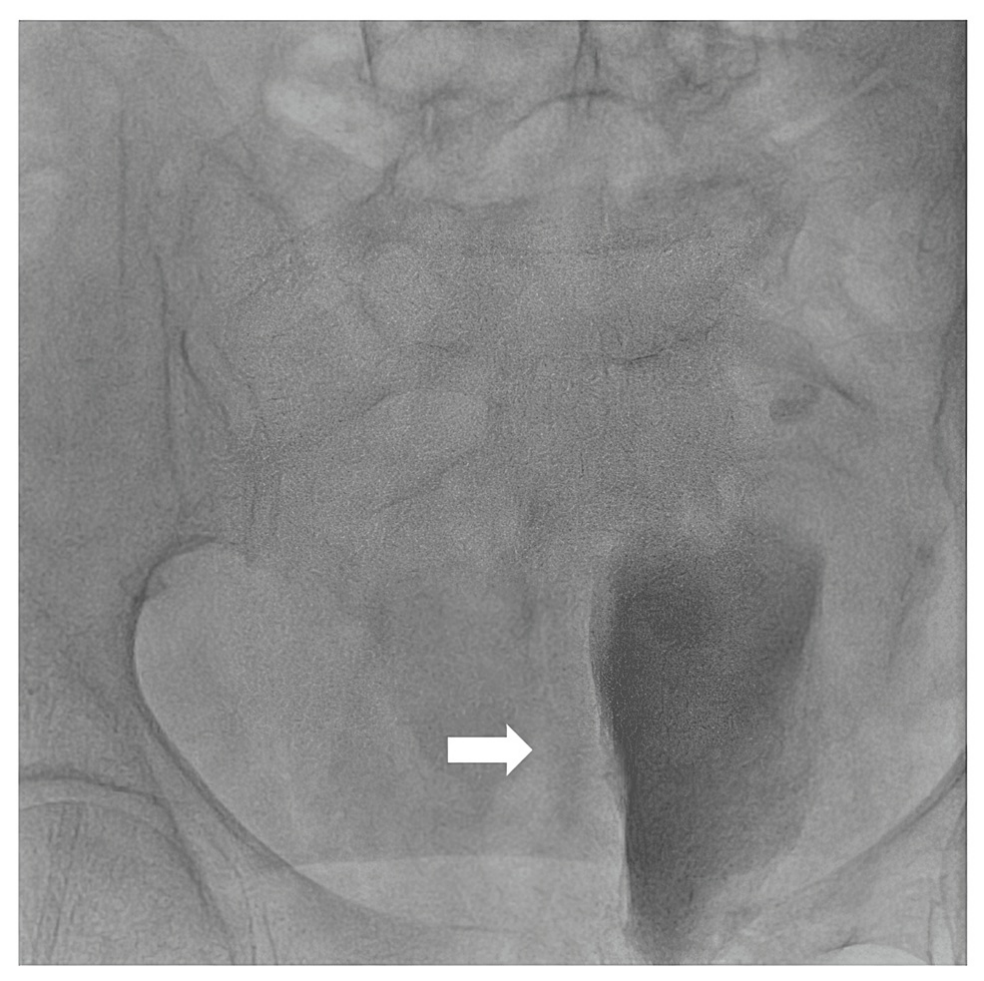
Bladder sign showing the contrast-filled bladder pushed to the left, implying a massive right retroperitoneal hemorrhage (arrow)

**Figure 3:**
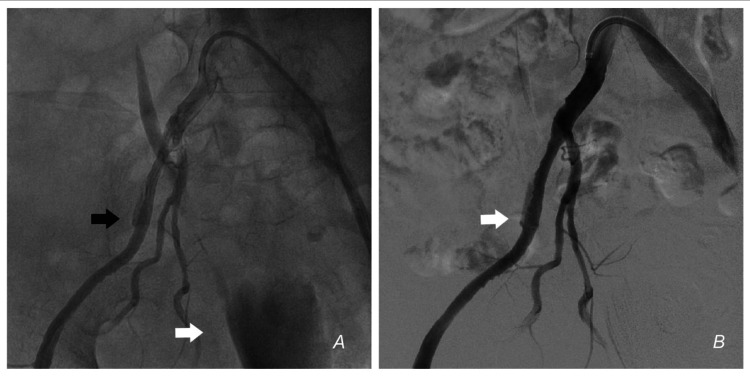
External iliac artery perforation (A) Injection in the right CIA from the LFA access shows the site of perforation (black arrow). The leftward shifted bladder can be seen (white arrow). (B) DSA. CIA – Common iliac artery, LFA – Left femoral access, DSA – Digital subtraction angiography

Inotropic support and fluids were started. Scout fluoroscopy revealed the bladder indentation sign - the contrast-filled bladder was pushed to the left, confirming the presence of a massive right RPH (Figure 2). The left FA was accessed, and a JR catheter was coursed over a wire across the left CIA and retrogradely into the right CIA. Angiogram and digital subtraction angiogram (DSA) revealed a perforation in the external iliac artery (EIA) with extravasation of blood (Figures 3A, 3B, Videos 1, 2). The perforation was distal to the origin of the internal iliac artery (IIA). Activated clotting time (ACT) was 265 seconds. We did not reverse heparin with protamine. An initial plan was made to control the bleeding with balloon occlusion. An 8 x 40mm peripheral balloon was inflated across the EIA, covering the site of perforation at 4 ATM for 1 minute, to stem the leak. Despite inflation for five cycles, there was a persistent leak and hemodynamic deterioration. Simultaneously, blood products were arranged, and transfusion started. As the perforation was away from the origin of the right IIA, we decided to implant a covered stent; however, there was no covered stent on the shelf.

 

**Video 1:** Angiogram from the CIA showing a perforation and extravasation of blood in the right EIA CIA – Common iliac artery, EIA – External iliac artery

**Video 2:** DSA showing the perforation DSA – digital subtraction angiogram

**Figure 4:**
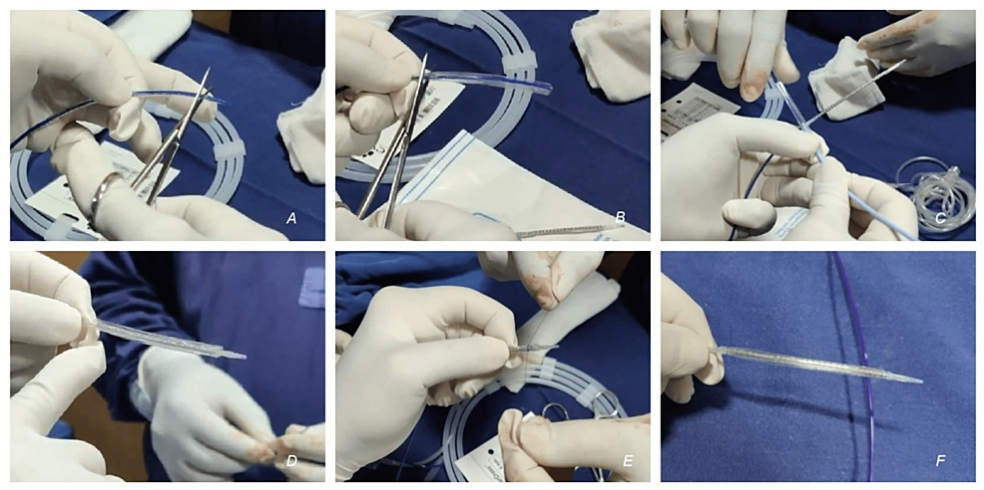
Preparation of covered stent (A) Removing the balloon material from the balloon using scissors at the distal end of the balloon catheter. (B) Proximally. (C) Using a dilator, the balloon material is removed from the shaft. (D) The balloon material is inserted over the peripheral stent. (E) Absorbable 5-0 sutures are placed proximally and distally to secure the two together. (F) The self-made covered stent.

So, a self-made covered stent was prepared (Figures 4A-4F - step by step, Supplemental Video 1). A 7 x 60 mm Admiral Xtreme PTA balloon catheter (Medtronic, USA) was taken. The proximal and distal parts of the balloon were cut from the shaft, and the sleeve of balloon material was removed. This balloon material formed the outer layer of the self-made covered stent. This balloon material was slid and mounted over a balloon expandable, rapid exchange 8 x 59 mm Omnilink elite stent (Abbott Vascular, USA). Two 5-0 absorbable sutures (Vicryl, Ethicon Inc, USA) were tied to the proximal and distal ends of the balloon material and the stent, firmly holding the two together. These sutures were placed to hold the balloon material to the stent (forming the covered stent) as it traverses through the catheter. As the covered stent is deployed, the sutures would break, sandwiching the balloon material between the vessel wall and the deployed stent.

**Figure 5:**
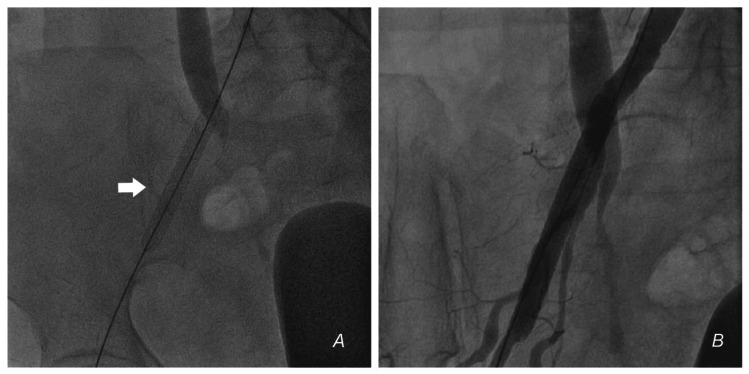
Deployment of self-made covered stent (A) The covered stent is deployed. (B) Angiogram after deployment showing no leak.

**Figure 6:**
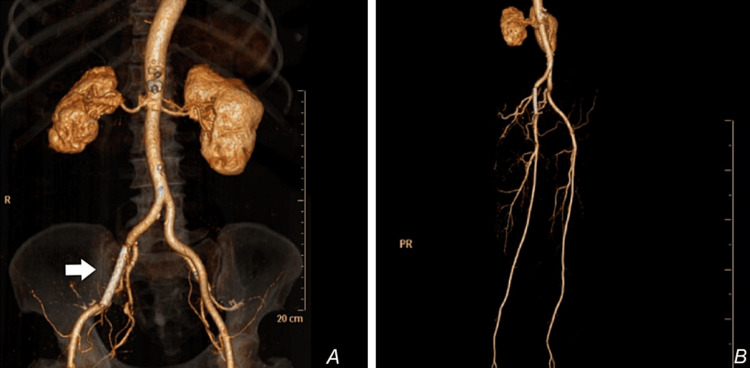
Post-procedure reconstructed CT angiogram of the lower limbs (A) Good stent position (white arrow) and no residual leak. (B) Good distal perfusion and patent stent.

This self-made covered stent was deployed in the right EIA after confirming the position angiographically. The sutures gave out at 5-6 ATM and the covered stent was implanted successfully. Angiogram and DSA were repeated, and they confirmed the successful sealing of the perforation (Figures 5A, 5B, Videos 3, 4). The patient was shifted to the CCU. Inotropes were weaned and she recovered well. Computed tomography (CT) lower limb angiogram done after five days showed good stent position and distal flow (Figures 6A, 6B). She was discharged on dual antiplatelets and rivaroxaban 10 mg/day. At one month, she was switched to a single antiplatelet (clopidogrel) and rivaroxaban was continued. She is doing well at six months of follow-up.

**Video 3:** Deployment of the stent The distal sutures give way at around 5 ATM during deployment. ATM – atmospheric pressure

**Video 4:** Post stenting angiogram shows no leak, and good distal run-off

 

A later review revealed that the J-tipped 0.035” Teflonwire, which was placed prior to advancement of the EBU coronary guide catheter had perforated the EIA and caused RPH. However, the patient continued to be hemodynamically stable during the coronary intervention.

## Discussion

Vascular complications following TFA are unavoidable with their use to facilitate complex coronary and valvular interventions. The incidence of RPH is less than 1% [3-5], and if not managed emergently, can lead to deterioration and death. The important cause of death is the delay in diagnosis. RPH occurs due to a few factors (Table 1) [3,5]. While dissections at the level of EIA are frequently encountered in diseased arteries, RPH following perforation in the EIA is uncommon. Perforations can happen in large arteries or their branches. Perforation of these internal, non-compressible arteries can cause rapid and uncontrollable extravasation of blood.

**Table 1:** Risk factors for retroperitoneal hemorrhage

Operator related factors	Procedure related factors	Patient related factors
High puncture	Anticoagulation and antiplatelets	Older age
Through and through puncture into the posterior wall of femoral artery	Use of GP IIb/IIIa inhibitors	Female sex
Puncture without ultrasound, fluoroscopic or angiographic guidance	Emergency interventions	Tortuous, calcified and diseased arteries resulting in guidewire related perforation.

RPH is suspected clinically when there is sudden hemodynamic collapse during PCI. Tachycardia and hypotension should prompt a search for RPH, after ruling out coronary perforation, cardiac tamponade, ostial coronary dissection and myocardial stunning. Immediate fluoroscopy of the groin and bladder will reveal the bladder indentation sign, which is specific for RPH, as the expanding hematoma compresses the contrast-filled bladder [6]. In a patient with normal renal function, the kidneys inevitably excrete contrast-producing pyelograms and cystograms. Contrast injection through the femoral sheath and DSA will reveal the site of the leak. Often patients are shifted to the CCU, and hemodynamic collapse is late, as the retroperitoneal space can hold a lot of blood before symptoms set in. Contrast CT can help diagnose such cases.

Once identified, fluids and blood products should be administered to replete lost volume. Contralateral femoral access should be obtained, and angiography performed to identify the site of the leak. Endovascular balloon tamponade from the contralateral femoral access will control the leak and provide temporary relief [7]. Small perforations may seal with prolonged low-pressure balloon tamponade and reversal of anticoagulation with protamine. Once temporary hemodynamic control is achieved, definitive endovascular management options include 1) coil embolization of perforated branch vessels, 2) local thrombin injections or thrombin blood patches [8] and 3) use of covered stents or stent grafts. Although surgical exploration and direct repair is the ideal management choice, in this subset of rapidly deteriorating patients, surgical mortality is high. Endovascular management has better short-term outcomes.

Deployment of a covered stent is the favored management option in this subset of patients. As we did not have a covered stent on the shelf, we fashioned a covered stent using easily available materials. This has been reported previously [9] and we have used self-made stents at our institute in cases of aortic coarctation during coarctoplasty with excellent long-term outcomes (unpublished data). Anticipated problems in the use of these self-made stents include 1) the presence of thrombogenic balloon material and 2) the unknown response of the underlying vessel with the potential to cause vascular calcification, atherosclerosis or aneurysms. In contrast, the commercially available covered stent systems have provisions to prevent these complications and have data on long-term outcomes [10]. But a self-made covered stent can be useful in an emergency when a covered stent is not available.

We continued the patient on a directly acting oral anticoagulant (DOAC), to potentially avoid thrombosis of the stented artery. Although no evidence is available for DOACs in this setting, anticoagulation in addition to an antiplatelet may potentially avoid thrombosis of the covered stent. At one year of follow-up, we plan to stop clopidogrel and continue DOACs alone. Mid-term outcomes at six months were excellent, with no symptoms of limb ischemia, with good Doppler signals distally. Our method of creating a self-made covered stent can be potentially useful in low and middle-income countries where commercially covered stents may not always be available. Securing the stent and the balloon material with absorbable sutures ensures that they stay together without migration until deployment.

## Conclusions

RPH is a catastrophic vascular complication following femoral access. Any intra-procedural or late hemodynamic compromise should alert the operator of a potential RPH. Endovascular management with rapid balloon tamponade followed by implantation of a covered stent is preferred over surgical repair. We describe our method of creating a self-made covered stent, its successful deployment, discuss potential problems and demonstrate good mid-term outcomes.
